# Predicting What Will Happen When You Intervene

**DOI:** 10.1007/s10615-016-0615-0

**Published:** 2017-02-07

**Authors:** Nancy Cartwright, Jeremy Hardie

**Affiliations:** 1 Durham University, University of California, San Diego, San Diego, USA; 2 Centre for Philosophy of Natural and Social Science, London School of Economics, Centre for Humanities Engaging Science and Society, Durham University, Durham, UK

**Keywords:** Causation, Support factor, Deliberation, Ex ante, Prediction

## Abstract

This paper offers some rules of thumb that practicing social workers can use for case studies that aim to construct, albeit not fully and never entirely reliably, models designed to help predict what will happen if they intervene in specific ways to help this particular client, here and now. We call these ’ex ante case-specific causal models’. ’Ex ante’ because they are for before-the-fact prediction of what the likely effects of proposed actions are. ’Case-specific’ because we are not concerned with studies that provide evidence for some general conclusion but rather with using what general and local knowledge one can get to predict what will happen to a specific client in the real settings in which they live. ’Causal’ because this kind of case study aims to trace out as best possible the web of causal processes that will be responsible for what happens. In this sense our case studies resemble post facto realist evaluations.

## Introduction

This paper offers some rules of thumb that practicing social workers can use for case studies that aim to construct, albeit not fully and never entirely reliably, models designed to help predict what will happen if you intervene in specific ways to help this particular client, here and now, and what will happen if you don’t. We call these “ex ante case-specific causal models”. “Ex ante” because they are for before-the-fact prediction of what the effects of proposed actions are likely to be, by contrast with “post facto” evaluation that tries to determine what was responsible for the effects that occurred or whether a particular action produced the outcomes intended, which are the meat of after-the-fact evaluation that aims to find out what went right, what went wrong, and why. “Case-specific” because we are not concerned with studies that provide evidence for some general conclusion but rather with using what general and local knowledge one can get to predict what will happen to a *specific* client in the real settings in which they live. “Causal” because this kind of case study aims to trace out as best possible the web of causal processes that will be responsible for what happens. In this sense our case studies resemble post facto realist evaluations. We take the causal processes to be real and recommend models that provide as realistic an account of them as possible, at least with respect to the dominant causes. But ours are ex ante not post facto and the case-study strategies we propose are meant to be responsive to that difference. Also, as you will see, just as with Pawson (2014), we caution against codifying what you should do since situations vary so widely.

Although we are writing in the *Clinical Social Work Journal*, the principles that ground the case study methodology described here apply across a range of social work settings and even more broadly, wherever we try to estimate what will happen when we act.

*The case study* is a broad church, so too is *case study methodology*. Case studies come in a great variety of forms, for a great variety of purposes, using a great variety of methods—including both methods typically labelled “qualitative” and ones typically labelled “quantitative”.^1^ As noted, our focus is on case studies that aim to establish causal conclusions, especially those linking social work interventions with the outcomes that may, or may not, result from them and on case studies intended to help explain what is producing problems in the first place.

It is often argued that causal conclusions require a comparative methodology. On this view the *counterfactual* is generally supposed to be the essence of causality: In situations where C and E both occur, “C caused E” *means* “If C had not occurred, then E would not have”.^2^ And it is additionally supposed that the only way to establish that kind of counterfactual is by contrasting cases where C occurs with those where C does not occur in circumstances that are similar to the first with respect to all other factors affecting E than the occurrence of C and its downstream effects. We maintain that neither of these suppositions is correct.^3^ There are arguments on both sides but we shall not rehearse them. Here we aim to focus on the needs of real practice. Whether or not comparative studies are ideal for warranting causal claims, we all regularly in daily life and in professional practice must bet on causal claims and guide our actions by these bets without the aid of comparison. Juries decide whether the defendant committed the crime generally without consulting a case just like this one but for the defendant’s actions; Nancy confidently infers that it was her second daughter (not the first, not her granddaughter, not Santa) who slipped *Northanger Abbey* into her Christmas stocking; and the NASA (United States National Aeronautics and Space Administration) investigating team decided that the failure of an O-ring seal caused the Challenger disaster. Social workers are in the same boat. You must draw causal conclusions and bet on what will happen for this client in this case, and you generally must do so without the benefit of a twin case to consult where you can observe what happened without the intervention you are considering.

We take it to be clear that each of these causal conclusions can be true or false and the reasoning and evidence that backs them up can be better or worse. We shall here discuss ways to make the warrant for them better, focusing on the kinds of causal conclusions that social workers engage with in each and every case, throughout the case. We believe that the advice we outline wears its plausibility on its face but we do not think that is enough. Plausible is, ceteris paribus, better than implausible, but it is better still when the proposals are also grounded in theory—plausible, well argued, well warranted theory. There is indeed theory to back up what we recommend, theory about both the nature of causality and of evidence, theory presupposed in a variety of scientific contexts and philosophically articulated and defended in detail in critically peer-reviewed venues. Of course, there is always scientific and philosophic dispute but the lessons we draw are relatively uncontroversial from a variety of different standard accounts of causality and of evidence for it. In particular, the theory we presuppose provides an underlying defense for many of conventional scientific methods of causal inference, from randomized controlled trials to qualitative comparative analysis, Bayes nets methods for causal inference, econometric instrumental variables, and others. You can read more about this in Cartwright and Hardie (2012).^4^

Much of the discussion in social work methodology and elsewhere about the advantages and disadvantages of case study methods for drawing causal conclusions supposes that the aim is to draw causal conclusions that can be expected to hold more widely than in the case at hand. This is not our focus. Our focus is the reverse. We are concerned with using knowledge that applies more widely, in consort with local knowledge, to construct a case study that will help you predict what will happen in the single case—*this* case, involving *this* client, *here* and *now*, but also *into the future* as things change in the client’s life and environment and in the provisions that it may be possible to provide.

Every reader of this journal knows that predicting what will happen if and when you intervene in this or that way—or if you don’t—is extremely difficult, and results are always uncertain. We want to underline this from the start. As a social worker, you know that you need actively to expect complications in predicting what will help your client. A lot of these have to do with what’s causing or might cause what. There is an ever present and bewildering variety of factors that might be causing the problem you are concerned with. Changes in circumstances (independent of any intervention by you) can affect your client’s welfare, create new problems or alleviate existing ones. And so can any of your possible interventions, which may work for one person in one setting but not for others or in different settings. In social work, you are not just concerned with predicting the consequences of your actions but also with predicting what is likely to happen in the life of the client anyway.

Simple domino models of causality can suggest that all this is far easier than we all know it to be. One straightforward way to get a grip on it is to construct a special kind of case study, one geared to constructing a case-specific causal model, laying bare the causal structure of what has been happening in your client’s life and what can be expected to happen under various interventions. In what follows we shall explain what a case study like this consists in.

Even when you have done the best you can at this, the basic lesson remains. Life is complicated and outcomes are hard to predict even if we do not adopt a simple domino model of causality but try to outline a more realistic model. It is important to recognize that the case study methods we describe can make predictions more reliable but that given the kind of general knowledge available and the local knowledge about your clients, their environment, and the supporting systems available that you are likely to be able to obtain, your models will be “rough-and-ready”—incomplete and not nearly as accurate as one would hope. So, it is important to expect that failure may occur and to be prepared to deal with it as best—and as early—as possible. Given this, it may be that the title of this paper should be amended, to—“Predicting *a little more accurately* what will happen when you intervene.” In the concluding section of this paper we will briefly discuss how, when it comes to reliability, this kind of case study methodology compares with other methods for predicting outcomes in the kinds of complicated settings that social workers generally face.

We propose then to give guidelines for conducting a case study that can help you to build a causal model geared to predicting what will happen when you intervene. Our purpose is to make explicit a kind of checklist to think through in constructing a causal model for your case. A perfect model will make perfectly reliable predictions. It is of course not possible to build a perfect model. But with the case-study methods we describe we believe you should be able to build better ones.

### What You Need to Do

When you are deliberating about the welfare of your client, you need first to form some understanding of what the problems are. Then you have to figure out as best you can what the causes of these problems are and what is likely to happen if you do nothing. Then you must propose some possible courses of action and consider what would happen under any of these alternatives. Finally, you have to evaluate the upsides and downsides and make a decision. So: Working out what will help the client you are dealing with requires you to draw on *your causal understanding of the client’s problems and on how any proposed intervention will work*. You need to.

1. know where your client’s problems lie,
2. work out as best you can what the causes of these problems are,
3. predict what is likely to happen if you do nothing,
4. propose some courses of action,
5. think through

- what would happen under these alternatives,
- what the costs and benefits would be,
  - for whom, and then,

6. decide the best course of action.

Though these are conceptually distinct requirements, the information that helps in fulfilling them can have significant overlap. For instance, in the United Kingdom providers of psychological therapies are encouraged to offer patients choice about their treatment. This may not only raise the chances that the intervention chosen succeeds,^5^ as in the case of Betty described below, but this may be of help in locating some of the causes of their problems by giving clues about their understanding of these causes.

In proceeding through these steps, you will be conducting a case study: a study of the causal structure of this particular case. It is worth noting that though “case study” and “causal structure” have a technical ring, this is the kind of thing you do all the time in everyday life, for instance in deciding whether to do the shopping before or after you pick up the children from school. There are no sure rules for how to do this. You must do your best to gather as much information from different sources as possible, including the clients themselves, to consult and review tentative conclusions with others, and to weave what you learn into the most coherent account of what is going on with your client that you can, and what you expect to happen if you intervene—or not—in various ways. There are no set procedures to follow in doing so to ensure you get it right—which is why continuing monitoring and review are so important. But we offer a 3-pronged strategy that can help.

This strategy is meant to help you focus on the different categories of information necessary for a sound case study that will allow you to diagnose the source of the problems and predict what might happen when you intervene. For concreteness, we will focus on the second stage: thinking about proposed interventions and their consequences. This involves an ex ante case study. But the strategies we offer for thinking about what will happen if an intervention is implemented can be turned to analyzing what are the sources of the problems to begin with—a “retrodictive” or post facto case study.

### Three Things You Need to Figure Out

We suppose that the Social Services in your area offer a number of alternative interventions you can call on that have proved to produce in some other settings results of the kind you and your client aim for in this case. For instance, if you are in the United Kingdom, NICE (the National Institute for Health and Care Excellence) has approved five different kinds of therapy for depression. So, if depression is an important feature in this case then helping your client to access free NICE-approved therapy on the National Health Service would be a good first step. This could be cognitive behavior therapy, counselling, interpersonal therapy, couples therapy, or brief psychodynamic therapy. Not all of these may be available in your local geographical area, as the latter three are in short supply. That is a starting point for your thinking—but just a starting point. We all know that the fact that an intervention has worked in some settings and for some people does not mean that it will do so for the person you are working with. If it is to help your client, there are three things, which we shall explain, that must be true, that you probably already recognize though perhaps do not set out in this direct way:

• The intervention must be *capable* of helping to produce the targeted result for your client.
• The *support factors* necessary for it to do so must be there—or you can arrange to get them there.
• Nothing must happen in the setting to overpower or derail the intervention.

As will become clear, dealing with these questions will require drawing heavily on your professional and practitioner experience.

### Is the Intervention Capable of Helping Your Client?

Thinking about this is a crucial first step in building your ex ante case study. Is there a realistic chance that this intervention can work for this person in these circumstances? Consider Betty who had a dual diagnosis—she had a substance abuse problem and a mental illness. In particular, she had problems with abusing alcohol and she struggled with depression. Betty was an outpatient with a United States local community health center. When she relapsed, the case manager who worked with her had made an early decision about interventions—not to call in the police. He could see that there was little chance that this intervention was capable of helping Betty. What did have a chance of succeeding, he judged, was working closely with Betty to understand and, if possible, remove or mitigate factors conducive to relapse and create positive incentives for her to stay sober. The case manager provides this narrative:

Betty did not like the fact that she was a client at the center. She was not openly hostile to me, and was in fact a very nice person, but she was court-ordered to work with the center and so was understandably resentful at having to do so. When I was first assigned to work with her, I mostly kept my distance. Betty had her own apartment and, since she did not have a mental illness that was so debilitating as to disrupt normal functioning, she worked various jobs to support herself. More importantly, she was maintaining her sobriety on her own through regular AA [Alcoholics Anonymous] meetings. Because Betty was taking care of herself in both of these regards, I would merely check in with her weekly to ensure that she was staying sober and otherwise doing well. Things remained this way until Betty relapsed. Once this happened, I met with her daily and talked with her extensively in order to help her.

One day I came by Betty’s apartment for my weekly check-in with her to find that she had started drinking again. Betty was used to her case managers calling the police on her when she had relapsed, which was part of the reason why she didn’t like working with the center. But I was not mandated to do this and I was not the kind of case manager that would do such a thing. I made it clear to Betty that I would not call the police on her and that, in fact, I thought it inappropriate to do so. I made it clear that I was really interested in talking to Betty to find out why she had relapsed. This was the first step in building trust with Betty and, more importantly, with helping Betty pull herself back into sobriety. Had I called the police on her as her previous case managers had done, this would have fueled her resentment to the center and would not have allowed Betty to pull herself back into sobriety. And pulling herself back into sobriety, as opposed to forcing sobriety on her, is what needed to happen here given how independent and in control of her own life Betty wanted to be.

An aid to thinking about whether an intervention has a starting chance—is *capable*—of working in a particular case is to recall that interventions don’t produce results by miracle nor by accident. When they work, it is for a reason: Because they do something. So the intervention should have some theory behind it. It needn’t be a deep theory. For instance, sending fathers to parenting classes might be intended to reduce their beating the child by teaching them other ways to affect the child’s behavior. But the theory *can* be deeper. It may draw on behavioral learning theory to explain why the fathers are responding to certain behaviors in their children (like crying or shouting) with violence, the intervention being aimed at altering this conditioned response. The job here is to understand why the intervention is supposed to work, and the clue is with the word *by*: It works *by* doing x. It is because the intervention can do *that*—for instance, teach fathers to respond in a less violent way when they hear the child crying—that the intervention can help produce the result you want.

Parenting classes can provide an example of one kind of reason, familiar from general social theory, that an intervention that has been successful in some cases and that is regularly recommended by United Kingdom social work teams (though less so in the United States, perhaps for this very reason) may not be capable of working in others cases. What seems like the same behaviors may mean very different things in different settings and different cultures. Consider making fathers attend parenting classes. Although this can lead to them learning better ways to deal with their children, there may be cultures in which being forced to attend these classes is seen as a public humiliation. The fathers go but don’t take in what they are being taught, they feel publicly belittled, and they go home and beat the child. Without substantial cultural change, sending fathers to parenting classes cannot play the role it is supposed to in this kind of setting. It is not capable of helping to produce the desired result there.

### What Support Factors are Needed?

Suppose your intervention can play the right role in your setting, it is capable of producing the right result. One thing to keep centrally in focus is that even if an intervention *can* work with your client—say, sending the fathers to parenting classes works by teaching them less harmful ways to respond to the child’s crying and there is no reason to think this father will not listen and try to take up some of the suggestions if he attends—still it may not actually do so because some of the other factors are missing that are necessary for the intervention to work as it should. You send the father but he can’t get there for the simple reason that the buses no longer run by the time he gets home from work.

The interventions you have available are seldom enough on their own to produce results. They need help, what we call “support factors”.^6^ Striking a match is a good way to get a flame. But not if it is sopping wet or there is no oxygen in the room. It is just the same with causes for social behaviors, or anywhere else for that matter. The cause you concentrate on is almost never sufficient by itself to produce the targeted result. There is always a whole team of supporting factors needed as well. If you don’t have these in your setting—or can’t arrange for them, or a good substitute, to be there—your intervention will not produce the intended result even if it is in principle capable of doing so. So, when you are considering an intervention, make sure all the support factors will be in place at the time. Think of support factors as necessary. If one is missing and you cannot find a substitute to put in its place, the intervention won’t produce the outcome you expect.

For instance, one mother we know of whose child was presenting behavior problems was referred to 6 different parenting programs, each one had approximately six sessions apiece, and she diligently attended all of them. Yet the problematic behaviors of her child persisted. It seemed the reason for this was the mother’s understanding of what was going wrong. She believed that the child’s behavior problems (like temper tantrums) indicated that he had a mental illness that needed treating. She didn’t see that what she did was playing a significant role in how her child behaved.

This case shows how parenting classes generally won’t work without the support of the parents’ belief that what they do is central in affecting the child’s behavior. And this is the kind of support factor that you can sometimes get into place, for instance by serious discussions with the parents about the relationship between a child’s behavioral problems and the behavior of the adults who interact regularly with the child. Though this is an essential step in any intervention’s success, social workers, especially newly qualified ones, may sometimes skip the explanation and checking-out step as if the intervention is self-explanatory.

For a more detailed example, here is more from the case manager’s narrative about Betty. Watch for all the places where support factors come into play. For ease of reference, we affix numbers in square brackets to factors we shall discuss.

By listening to Betty, I learned that her relapse was ultimately due to the fact that she found her life boring. One day she was working at a temporary job and, when her shift was done, a fellow employee asked if she wanted to smoke some marijuana with him. According to Betty, her life was really boring because it consisted mostly in AA meetings and working menial jobs to pay for rent and other necessities, and so, to spice up her life, Betty agreed to smoke marijuana with her fellow employee. Unfortunately, smoking marijuana usually led to her drinking, and that is indeed what happened in this case.

She wanted to get sober, but she did not want to go to rehab—she wanted to get sober on her own. I [1] respected her wishes here, and this, like [2] not calling the police on her initially, was crucial for [3] building trust with her and for respecting her autonomy. In fact, at this point I validated her desire to get sober on her own by making it clear to Betty that she indeed was the one in control of her life, and so she was the one that needed to make her sobriety happen. No one else could do it for her.

Then, after making it clear that she was in control of her life and her sobriety, I engaged in what is called “motivational interviewing” to see what might motivate Betty to get herself sober. It is at this point that I learned a very important fact about Betty: She had a daughter that she gave up for adoption years ago, which was a source of her depression. She did not think she had made the wrong choice in giving up her child for adoption, but she missed her daughter and really wanted a relationship with her—a relationship that she currently didn’t have. Betty had some minimal contact with her daughter over the years, and even had pictures of her up in her apartment, but Betty did not have a real relationship with her daughter. At this point, Betty’s daughter was still a minor living with her foster parents, and so Betty had to wait until her daughter was a legal adult until trying to forge a relationship with her. But this, however, did not really matter because [4] Betty had a deep desire to have a relationship with her daughter, and it was this that I focused on to motivate Betty to get sober. She was to think about the future relationship with her daughter that she wanted more than anything, and especially about the fact that she must be sober in order to build the relationship.

Besides locating a goal strong enough to motivate Betty to get sober, I also had conversations with her to find out [5] what she likes to do with her time. This was intended as another source of motivation to get sober—getting sober would allow her to do what she likes to do with her time instead of drinking heavily—as well as something to help her down the road to maintain her sobriety once she got it back. One thing that Betty really liked to do was arts-and-crafts. Unfortunately, Betty did not have [6] a table on which to work, and she could not afford one because she was poor. In response to this, I sent out a mass e-mail to my colleagues to see if anyone had some kind of table to donate and, before long, someone offered a free card table. I took it over to Betty’s apartment to give to her, and though she had not yet pulled herself back into sobriety, she at least had the table there for when she had.

Fortunately, Betty was able to pull herself back into sobriety. I cannot say how long this lasted because I did not work with Betty for much longer after her success, but she was able to get sober on her own, as she had wanted to do from the beginning of her relapse. Hopefully she was able to stay sober this time given that *she* made it happen.

In this case [1], and probably [2], were essential for [3] “building trust”, which in turn was essential for the motivational interviewing to unearth the kinds of things that could genuinely succeed in helping motivate Betty. [4]—the “desire for an eventual relationship with her daughter”—was a support factor to couple with Betty’s desire to be sober which had proved insufficient on its own to carry her through. [5] was another support factor that was probably essential for that outcome given that part of the cause of her relapse was boredom arising from having few activities she enjoyed, and [6] a simple support to allow her to take up the arts-and-crafts she liked.

In sum:

Trust required [1], [2] to support the efforts of the case manager.

Unearthing motivations required [3] to support the motivational interviewing.

Staying sober required [4], [5] to support Betty’s desire to stay sober.

Relieving boredom required [6] to support Betty’s interest in arts-and-crafts.

The same way of thinking can also be helpful earlier, when you ask not whether an intervention will produce the envisaged outcomes but rather ask what is causing the problem in the first place. If you think a particular cause is partly at fault for a problem you have identified, you can check to see if the requisite support factors are there since, if they are missing, it cannot be this cause that is producing the effect after all. If the requisite support factors are not there, look elsewhere for the causes.

Here are a few important things to keep in mind about support factors, many of which will be familiar. The first is that although almost every intervention requires support, there is often more than one way to provide it; there is more than one set of factors that can couple with the intervention to produce the same outcome. This is helpful to keep in mind since sometimes a support factor you have identified is missing for an intervention that you might like to try. Yet maybe there is a substitute that you can get into place that will do the same job in supporting the work of the intervention. For a simple straightforward example, one support factor for an intervention might be communicating in English with the service user. This may not work with some people, so providing interpreters can be an alternative way of achieving the goal of communicating.

Second, “there are more ways than one to skin a cat”. More generally, there is almost always more than one way to produce a given result—more than one set of factors that working together makes the effect likely. This is all too familiar in social work, where many individuals will suffer more than one set of factors, each by itself enough to create the problem you are concerned with. This is illustrated again by the case of Betty where multiple complexes of causes were at work. Her depression and her drive to drink were fueled by boredom, which stemmed from her life of AA meetings and menial jobs. Also, while her desire to have a relationship with her daughter ended up serving as a powerful motive for her to get sober, her on-going awareness of the distance between her and her daughter more than likely exacerbated her depression and her drive to drink.

The good news is that the same generally holds for interventions. There is often more than one thing you can do to relieve a problem—supposing of course that all the support factors are in place for each of these. This is familiar but sometimes we can lose sight of it when we are frustrated that a particular intervention we would like to use is not available or, irritatingly, that the intervention is available but not all the required support factors are there.

#### One Warning About Multiple Causes

Suppose your case study identifies a number of different sets of factors operating together in the same situation, each set likely to be enough on its own to produce the result. We may be tempted to think that their influence is in a sense “additive”: With two sets of causes each for the same effect, you may expect to get double the effect. But that is often not so. Sometimes two different sets of factors can interact in a way that heightens or lessens the effects of each. Or they may together have no effect, or even an effect opposite to the one each set would have on its own, a phenomenon known as “reversal of effect direction”.

This is something to keep in mind when you want to predict the effects of interventions. The reversal of effect direction can happen, for example, when several services become involved in providing different types of help with a cumulative impact not of strengthening the client as intended but of disempowering them to the point that they fail to make progress in learning how to cope on their own or of confusing them because they don’t know what is most useful or effective to focus on first, since different demands or recommendations from different agencies are not always well-coordinated. It is fairly typical for the overall effect of two interventions deployed together to be considerably less than the sum of what each can be expected to produce on its own. For instance, if two different services provide a supportive intervention, they may play more or less the same role, so that introducing one when the other is already in place may not produce much added value.

A third thing to note is how much we are likely to miss out on. No matter how knowledgeable we are and how carefully we build our ex ante case study, we will seldom be able to identify enough factors to ensure the outcomes we aim for. At best, we can hope to understand what will make an outcome more likely. So we should not overbid our cards. Sometimes the language used can make this lesson easy to forget. We hear talk of “causes” of the client’s problems and of “what works” to prevent or alleviate these problems. It is better to remember that good interventions may be conducive to the results you want but that even the best, most effective interventions may fail much of the time. Think, for example, about the Family-Nurse Partnership, which was developed in Colorado by David Olds and associates and which is being actively imported into England from the United States, where it claims rigorous evidence for success as a preventive early childhood program. Though Olds’ (2006) first evaluation of the program reported effectiveness overall, that overall result covered significant improvement for low income families and no significant improvement for wealthier families.

Fourth, even the phrase “helping to produce targeted results” may be over-optimistic. An intervention that is helpful or neutral for most people may, for some, contribute to a worsening of their problems. This means that there are support factors that couple with the intervention to produce beneficial results and there are support factors that couple with it to produce negative results, and very often we don’t know what either of these are. We are all familiar with the diversity of people’s responses to drugs with some having very adverse (even life-threatening) reactions to a chemical mix that is beneficial for most people. The same diversity of response occurs in social and psychological interventions. For instance, in many clinical trials a percentage −5 to 10 % according to Lambert and Ogles (2004)—of those given the “successful” treatment leave the trial worse off than when they came in; and in care on the ground, these figures can be worse.

### Will Your Intervention Get Derailed?

It is clear that however conscientiously you analyze your problem and think through what to do, you may not get the result you expect. This can happen in several ways that a good ex ante case study will try to take account of. Again, even our best efforts to foresee these and build them into our predictions will often fail. So monitoring, review and hedging your bets is essential.

#### Interruption

The process may be interrupted. Things may be progressing just as hoped for when, unexpectedly, something happens to put a stop to it. For instance, from child welfare, the mother’s violent ex-boyfriend shows up out of nowhere and moves back in. Or, here’s another case from the same community center that Betty was associated with, which supplied special sheltered housing. A few of the residents had problems maintaining their housing due to an inability to say no to homeless “friends” who wanted to crash with them, which was forbidden: One of the requirements was not to allow people to spend the night. For example, one client—like many from that community center—would go to the town center to eat free meals served to the homeless and the poor, which inevitably led to her letting her homeless “friends” stay with her despite the repeated reminders from her social worker and other center staff that this was against the rules and, more significantly, put her housing situation in jeopardy. This on-going issue almost got her evicted; she and her payee had to fight in court for her to keep her housing.

But we shouldn’t always think in terms of interruptions as intrusions that drop in from outside to block the route to improvement. Sometimes what happens is that a factor that you had rightly picked out as necessary and reasonably identified as likely to be present, and indeed present at the start, disappears. For example, again from child welfare, the grandmother who was giving the children breakfast and getting them off to school quarrels with the mother and stops coming. Or consider Betty again. After she regained sobriety she was having a hard time coming up with rent due to her recent relapse. Fortunately, her recovery was not derailed because she ended up getting help from a friend so that she didn’t get evicted from her apartment. But if she had been evicted and ended up homeless again this would have constituted a significant setback for her, which would have made it much harder for her to maintain her sobriety and most likely would have exacerbated her depression.

#### Offsetting

The beneficial effect of your intervention on the achievement of your target may be offset, overwhelmed even, by bad effects on that same target from other sets of causes which you had not foreseen. Your intervention is indeed a member of a set of factors that works to the good, e.g. all the necessary support factors are present, and indeed in a sense it does work, but the positive results are not visible because other factors have been present with an even stronger negative impact. You may have achieved a kind of “counterfactual” success: Matters would have been even worse had your intervention not been at work. On the other hand, if an intervention is too weak in the face of the negative effects, it may not be worth implementing. Put your efforts into looking for some alternatives.

#### Self-Defeat

Your intervention may have good results by one route but negative effects on the very same target by another route. For instance, many of the things social workers do to help parents can at the same time make them feel dependent and unable to act for themselves. So the intervention itself produces a negative effect on parenting as well as a good one. And, of course, you must always watch out for bad side effects of what you do. You may improve the targeted outcome but the situation gets worse overall because of the negative side effects of your intervention. The danger is not just that your intervention may fail, costing not only lost resources but also dashed hopes. The danger is also that it may do genuine harm.

This problem is especially likely with interventions that are social in nature since other people almost always have the potential to counterbalance an intervention’s positive effects. For example, social workers at Betty’s community center encouraged clients living in their sheltered housing to get out of the house and socialize with others, especially because they tended to have schizophrenia and depression, which tend to cause social isolation, which in turn exacerbates these illnesses. They were encouraged not only to attend social groups at the community center but also to get out into the community on their own and participate in community activities that they would enjoy. Unfortunately, this would lead them to places, such as the community “Drop-In” center for homeless individuals, where negative influences could often be found. For instance, we have already mentioned the negative influence of homeless “friends” wanting to stay with clients and thereby putting their housing in jeopardy. Another major negative influence that other people routinely had was to facilitate the substance abuse issues that the center was trying to combat. For instance, one of the clients had schizophrenia, problems with alcohol, and had the mental age of a 7-year old, and she would consistently let “friends” stay with her even though they would not only jeopardize her housing, but would facilitate her drinking and would even introduce her to other substances (e.g., crack cocaine).

#### Conclusion

We need to make clear that following these guides for constructing an ex ante case study cannot guarantee success, nor even guarantee that what you do achieves the best possible chance of success. It is a commonplace in the statistical literature and practical guides which use those insights that we cannot expect certainty and that any interventions we propose will have uncertain outcomes. It is often suggested that this uncertainty can usefully be represented in terms of probabilities. For instance, if an intervention requires the presence of three support factors, then we are to attach probabilities to their presence and hence calculate the odds that the intervention will succeed. This way of thinking aims to achieve a precise measure of the uncertainty about an intervention’s success.

But the problem of uncertainty is worse than this, and that fact has to be faced. It is not just that we find it hard to attach probabilities to the presence of the support factors. More seriously, we don’t fully understand how to answer the two key questions about causal roles and their required support. We are always in danger of misunderstanding how things work and how they will work if we intervene and of not taking into account events that we simply failed to anticipate and see the future significance of. These uncertainties, doubts, imaginative failures cannot be summarized in a single number for the probability of success. Two most likely consequences of over-estimating our ability to predict what will work are that time and money will be spent on services that have disappointing results and that professional social workers may become too confident and assertive in telling clients that they know what is best for them, thus further disempowering many who already have low status in society.

This is not a counsel of despair. Certainly, you can often decide to intervene with a good chance of success. But, as you recognize, you must also expect failure and so monitor progress and plan in advance to deal with failure if that is what you get. Equally important, you must not expect our suggestions, let alone techniques such as decision trees or cost benefit analysis, to provide the certainty which you would like. In difficult cases, those high levels of certainty are not available, nor even clarity about probabilities.

In the face of our many cautions about the difficulties and chance of failure using the case study methods we have described, you may reasonably wonder if some other methods for predicting the outcomes of proposed actions might work better. That’s an open question. What we suggest could be characterized as a “frontal attack”. We suppose that there is a network of interacting causal factors that will bring about whatever outcomes occur in your particular case. Our case-study strategy is designed to help you get an understanding of what these will be and how they will operate, as best you can. Sometimes you may just be too ill positioned to do this. There are too many unknowns and you don’t know how to go about learning what you need to know to make predictions that are at all reliable. What might you do instead?

If there’s an intervention that is available that has had good outcomes in many cases that at least superficially resemble yours, and it is not costly, and it does not seem likely to have bad side effects, recommend it. That seems to be the thinking behind the “stepped care” recommendation of the United Kingdom’s National Institute for Health and Care Excellence to try “low intensity interventions”, based usually around cognitive behavioral therapy principles of change, as the first line of treatment for mild to moderate depression.^7^ This can be a good strategy—so long as you remember that not all costs are financial and unwanted side effects are not so unusual. (For instance, there is evidence suggesting that, for many people, failed therapies leave them more depressed and less willing to continue to seek help.^8^) This strategy is in line with one standard piece of advice: When you don’t know anything about your case, assume it will behave as the average in cases that are at least superficially similar.

An alternative is to go along with the client’s preferences, or at least to use this as a starting point. As we have mentioned, there is some evidence that “routinely assessing and meeting patient preferences may improve the outcomes of psychological treatment” (Williams et al. 2016, p. 4) and this can give some clue to their understanding of the causes of their problem. They might not then select the right option but if you elicit their stated preference you can perhaps guide them to agree with you to what seems a better option (which may be a nearby neighbor intervention).

Which strategy then should you use? We can only conclude with our refrain throughout. What strategies you should use for prediction depend on what you know or can come to know. You can do better and worse at gathering and assessing this information. But no matter what strategy you adopt and no matter how well you think you’ve done at it, outcomes will always be uncertain so plan as well for what to do if things start to go wrong.

## Footnotes

1 For a nice discussion of case study types see Morgan (2014); for a good text surveying methods see Byrne and Ragin (2009).

2 See, for example, Menzies (2014).

3 For reasons why, see Cartwright (2015) on evidence for judgments about causes in the single case.

4 For a different, very formal approach that has the same broad lessons see Imbens (2004).

5 See, for example, Williams et al. (2016).

6 The importance of these has been argued in philosophy by JL Mackie (1974), who explains that what we generally call “the cause” is usually only a necessary part of a set of factors that must act together to produce the effect. This role is widely recognized in the social and medical sciences where these support factors are called “moderator variables” or “interactive variables”. Epidemiologists represent the total set of factors that cooperate together to produce an effect in a pie diagram. You may notice that the categories we describe are not firmly differentiated. Perhaps punitive measures would have worked for Betty had she been a different kind of person with a different history. Should one think of “not having Betty’s history” as a support factor instead of saying, as we did, that punitive measures didn’t have a starting chance with Betty? There’s no cut and dried fact of the matter. But it is usual to take support factors to be features that could reasonably be changed in the case at hand.

7 See the National Institute for Health and Care Excellence guideline on depression (2009) and the Bower et al. (2006) review this is based on.

8 See, for example, Parry et al. (2016).
